# Haemovigilance in India during the COVID-19 pandemic

**DOI:** 10.7189/jogh.13.03030

**Published:** 2023-09-22

**Authors:** Rati Sudha, Isha Goel, Poonam Katiyar, Gauri Misra, Reema Roshan, Naveen Sharma, Saurabh K Sharma, Amit Katiyar

**Affiliations:** 1 P.G. Department of Zoology, ANS College, Magadh University, Patna-803123, Bihar, India; 2 ICMR-AIIMS Computational Genomics Center, Indian Council of Medical Research, Ansari Nagar, New Delhi-110029, India; 3 NCC-Pharmacovigilance Program of India (PvPI), Indian Pharmacopoeia Commission, Raj Nagar, Ghaziabad-201002, Uttar Pradesh, India; 4 ESI Dispensary, Employees State Insurance Scheme, Labour Department, Government of Uttar Pradesh, Mohan Nagar, Ghaziabad-201007, India; 5 Molecular Diagnostics and COVID-19 Kit Testing Laboratory, National Institute of Biologicals, Noida-201309, Uttar Pradesh, India; 6 International Health Division, Indian Council of Medical Research, Ansari Nagar, New Delhi-110029, India; 7 Bio-Medical Informatics Division, Indian Council of Medical Research, Ansari Nagar, New Delhi-110029, India; 8 School of Computer and Systems Sciences, Jawaharlal Nehru University, Mehrauli, New Delhi-110029, India; 9 Bioinformatics Facility, Centralized Core Research Facility, All India Institute of Medical Sciences, Ansari Nagar, New Delhi-110029, India; * Joint first authorship.; † Joint senior authorship

## JOURNEY TO SAFE BLOOD

Critically ill patients who have lost blood must receive blood transfusions in order to survive, but could encounter adverse effects during this process. Any adverse incident that occurs during or after a transfusion of whole blood or one of its components and is not associated with any other factor is considered a transfusion response. Most of these adverse reactions are non-infectious in origin and can occur suddenly or gradually. It depends on the severity and appropriate therapeutic intervention whether adverse events are minor, moderate, severe, or life-threatening [1].

To prevent medical malpractice during blood transfusion by practitioners anywhere in the world, the medical protocol or transfusion medicine known as “Haemovigilance” is followed [2]. The International Haemovigilance Network (IHN) and the International Society of Blood Transfusion (ISBT) define haemovigilance as a surveillance system for blood donation, collection, storage, and transfusions, allowing adverse reactions to be recorded and evaluated to correct their causes and prevent recurrences, thereby protecting patients' safety [3,4]. The first hemovigilance systems were established in Japan in 1992, followed by France (1994), Germany (1994), Greece (1995), Luxembourg, and the UK (1996) [5,6]. According to the World Health Organization (WHO), 128 countries have a national health surveillance system for the appropriate clinical use of blood, of which 32 countries are in the African region (74% of reporting countries), 23 are in the Americas (70%), 12 are in the Eastern Mediterranean (67%), 33 are in Europe (80%), nine are in South East Asia (90%), and 19 are in the Western Pacific (76%) [7]. The haemovigilance system varies from one country to another, and globally, approximately 107 million units of blood are donated and collected annually, of which 50% comes from developed countries, where the rate is 39.2 donations per 1000 population, and the rest from developing and underdeveloped countries, with 12.6 and 4.0 donations per 1000 population, respectively [8]. The majority of blood transfusions in low-income countries are given to children under five years of age; however,76% of all blood transfusions are administered to patients over 60 years of age in high-income countries.

Most developed countries have defined their haemovigilance systems; in the UK, it’s called “Serious Hazards of Transfusion” (SHOT), while in the Netherlands, it’s called “Transfusion Reactions in Patients” (TRIP). A similar system exists in Canada, known as the “Transfusion Transmitted Injuries Surveillance System” (TTISS) [9]. In contrast, BRICS countries (Brazil, Russia, India, China, and South Africa), represent over half of the world's population, are still struggling with their health monitoring systems, especially during COVID outbreaks. A National Hemovigilance System (NHS) was officially implemented in Brazil in 2002, starting with a network of 100 tertiary hospitals with voluntary reporting of all reactions. In 2009, the reported rate of reactions was 0.91/1000 transfusions [10]. According to the Chinese Haemovigilance Network, founded in August 2017, the adverse reaction rate associated with blood transfusions in 2019 was 0.2%. The situation is grim in Africa that still lacks an effective haemovigilance system [11]. India also lacked an effective and standard system for reporting an adverse transfusion event until 2012, when with a gradual increase in awareness, a centralized haemovigilance programme for India was established [12]. The number of adverse blood transfusion reaction reports submitted to HvPI continues to rise since the launch of “Hemo-Vigil” in January 2013. There were 953 TRs in 2013, 1144 TRs in 2014, 1372 TRs in 2015, 1521 TRs in 2016, and 2792 TRs in 2017 [13]. Among transfusion reactions, the most frequent is Febrile Non-Hemolytic Transfusion Reaction (FNHTR), which occurs in 40.84% of cases. Mild allergic reactions category comprised 27.26% of the reactions. Anaphylactic/hypersensitivity reactions were 12.68% and hemolytic transfusion reactions were 4.31%. Other reactions reported to HvPI include TAD: Transfusion Associated Dyspnea (2.38%), TACO: Transfusion Associated Circulatory Overload (0.67%), PTP: Post-Transfusion Purpura (0.64%), TTBI: Transfusion-transmitted bacterial infection (0.46%), TRALI: Transfusion Associated Acute Lung Injury (0.26%); TAGvHD: Transfusion-Associated Graft Versus Host Disease (0.03%). In September 2020, India had overtaken Brazil as the country with the second-highest number of Coronavirus cases (4.2 million). Despite no reports of trans-fusion-transmitted coronaviruses, including SARS-CoV-2, worldwide as a result of the COVID-19 pandemic, this outbreak imposes many unprecedented challenges to the routine care of patients, including those with Thalassemia. The COVID-19 pandemic had affected transfusion services in India, and therefore national guidelines for blood transfusion in India were formed.

## HAEMOVIGILANCE PROGRAMME OF INDIA

**Figure 1:**
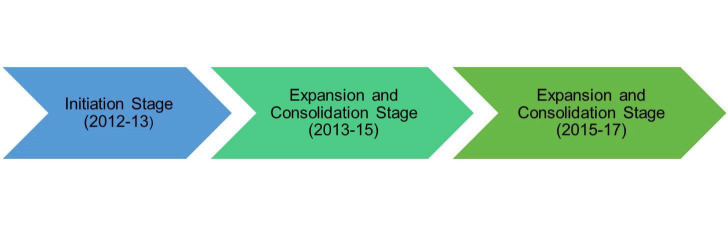
Five years (2012-2017) of HvPI development stages.

The Haemovigilance Programme of India (HvPI) was launched on December 10^th^, 2012 at the national level in 90 medical institutions across the country by the National Institute of Biologicals (NIB), Ministry of Health and Family Welfare, Government of India as the National Coordinating Centre (NCC) under its Pharmacovigilance Programme of India (PvPI); it encompassed 90 medical institutions across the country [14,15]. This provision came under the 12^th^ five-year plan 2012-2017 with a budget of ₹293.6 million and was divided on a yearly basis into three stages of development (**Figure 1**). For the first financial years of 2012-2013, the focus was on the establishment of the system, the development of the protocols, creating awareness through zonal workshops/newsletters, and also the enrolment and collection of data from the institutions. The second stage between the years 2013-2015 was for the expansion and consolidation where the focus was on training the staff, getting membership of the International Haemovigilance Network (IHN) and, simultaneously, conducting more zonal workshops, publishing newsletters, enrolling new members and data collection. The final stage from the years 2015-2017 was for expansion and maintenance, where more initiatives were taken for system maintenance, training, evaluation of the donor vigilance viability, and the planned development of epidemiological surveillance for transfusion-transmissible infections (TTIs) [16,17]. By the end of December 2021, HvPI had enrolled 1190 out of a total of 2760 licensed blood banks in the country, including the recent addition of 391 new blood banks during the COVID-19 pandemic.

### Structure and hierarchy of the HvPI

**Figure 2:**
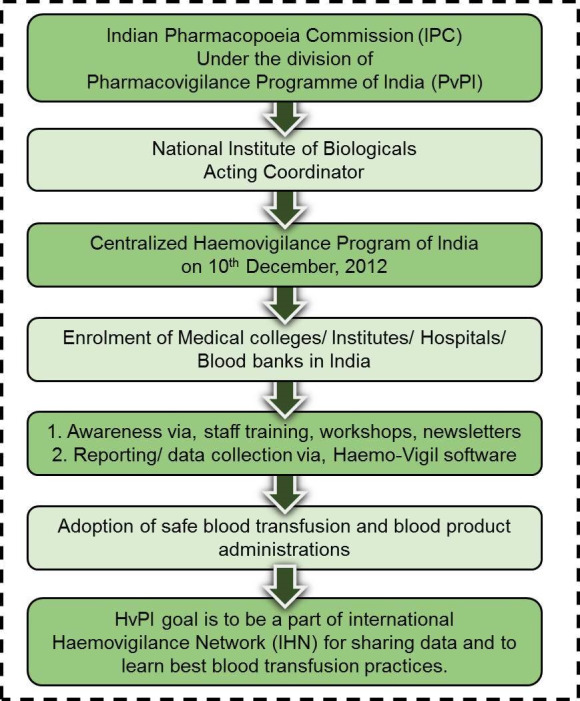
Structural framework of Haemovigilance Programme of India.

The National Institute of Biologicals (NIB) serves as the HvPI coordinating centre, collecting, combining, and analysing country-wide data on biologicals and haemovigilance across the country (**Figure 2**). The “Haemo-Vigil” software of NIB provides a user interface for reporting adverse reactions through the head of the enrolled institutions. Ultimately, HvPI aims to help health practitioners adopt safe blood transfusion practices by tracking and recording adverse reactions related to blood transfusions and blood product administrations. Further, HvPI has set its sights on joining the International Haemovigilance Network (IHN), which currently has 28 members, to provide a platform where haemovigilance data can be shared internationally to improve learning and benchmarking of best blood transfusion practices.

### Objectives of the HvPI

**Figure 3:**
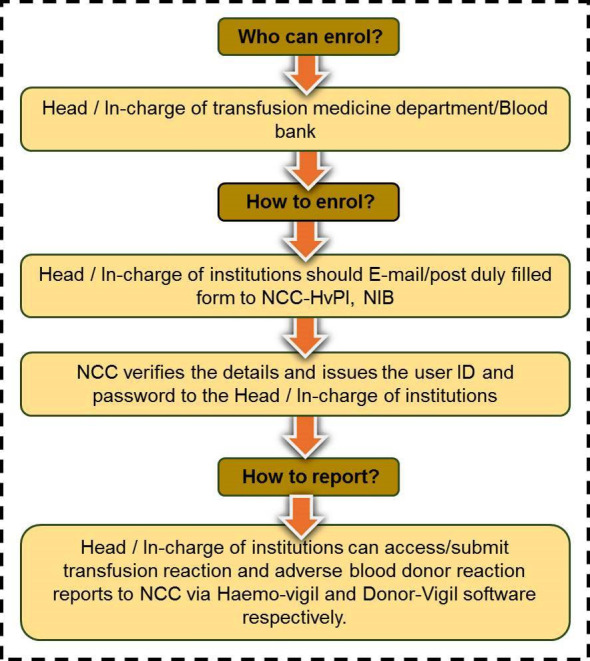
Objectives of HvPI.

The haemovigilance advisory committee drafted a document-based guideline for collecting and analysing adverse transfusion reactions [18]. A haemovigilance program primarily aims to detect and analyse all untoward effects of blood transfusions to prevent recurrences. The detail objectives of HvPI include (i) monitoring Adverse Drug Reactions (ADRs) and the risk-benefit ratio of medications in the Indian population, (ii) educating healthcare professionals about best practices for Haemovigilance and raising awareness of the programme, (iii) keeping stakeholders up to date on the latest findings, (iv) assisting the Central Drugs Standard Control Organization (CDSCO) in formulating safety-related regulatory decisions for medicines, (v) offering independent, evidence-based approvals regarding the efficacy of medications, (vi) establishing a national centre of excellence that meets international standards for drug safety monitoring, (vii) creating a platform for communication and knowledge exchange via the International Healthcare Network (IHN) and are summarized in **Figure 3**.

### Features of the HvPI

**Figure 4:**
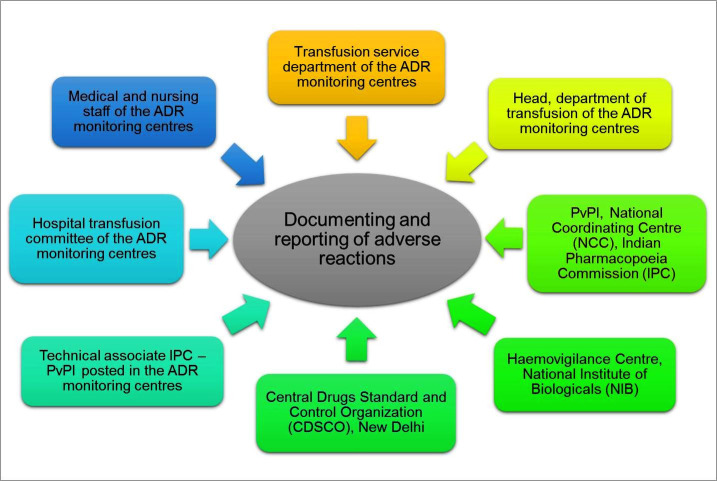
Coordination and collaboration among departments involved in the reporting of blood transfusion-related adverse reactions.

So far, the HvPI has received a positive response from institutions across the country, with many of the have already enrolled in this programme. **Figure 4** depicts the relationship between the various departments involved in the documentation and reporting of blood transfusion-associated adverse reactions. Head/In-charge of the respective transfusion department can submit the Transfusion Reaction Report and Adverse Blood Donor Reports to the NCC. The transfusion reaction workup form is used to document all signs and symptoms, transfusion and transfusion product details, and workup on reaction details. The TR-TD (Transfusion Reaction Traceability Document) form and these records are to be maintained by the Department of Transfusion Medicine or blood centres. The transfusion reaction reporting form (TRRF) for blood/ blood components/ plasma products is used to notify the HvPI, NCC, and NIB of the reaction. Various departments work collaboratively to record and store all data for the NCC and NIB using the Haemo-Vigil software. The Haemovigilance Advisory Committee (HAC), which is made up of clinical experts trained to identify underlying causes, evaluates these reports.

### Awareness programmes conducted by the HvPI

The success of any programme depends on the level of awareness created among the relevant stakeholders. The first awareness programme under the PvPI was pre-sented at the national conference of the Indian Society of Transfusion Medicine on 24 November 2012, during a session on "Haemovigilance". Since then, workshops, symposiums, training, newsletters, and scientific publications have been held or published regularly throughout the country as a part of continuing medical education (CME). The CME contents have been prepared with the following objectives: (i) creating awareness about the program; (ii) providing information on what is re-portable, how it is defined and documented; and (iii) demonstrating how to upload reports using Haemo-Vigil software. In addition, a brief communication about the launch of the HvPI was published in the official journal of the Indian Society of Transfusion Medicine [19]. NIB has recently hosted a study tour for Bangladeshi delegates on capacity building on the Haemovigilance system from 8 to 12 October 2018. The goals of this visit were to first understand the Haemovigilance protocol for blood donors and patients and to see how it is implemented at NIB; second, to learn how data from blood donors and patients is compiled and reported; and finally, to learn about the use of data/reports for the prevention of adverse events. The Haemovigilance Newsletter (soft and printed copy) is being published on a biannual basis by the National Co-ordinating Centre. The above steps are being taken to sensitise and encourage the participation of healthcare professionals in the country.

## SCOPE OF HAEMOVIGILANCE

Haemovigilance covers the entire transfusion chain, starting with blood collection and continuing with follow-up. All aspects of the process are coordinated by the blood transfusion services, hospital clinical staff, transfusion laboratories, hospital transfusion committees, and regulatory agencies. The COVID-19 outbreaks have severely impacted both the donation and supply of blood and the management of clinical transfusions. By adopting flexible policies, preparing emergency plans on time, ensuring timely and accurate communication with regulatory and public health institutions, ensuring blood collection meets clinical needs, ensuring staff and donors' safety, and minimizing virus transmission through blood transfusions, the HvPI may ensure timely and accurate communication with regulatory and public health institutions.

## NATIONAL GUIDELINES FOR SAFE BLOOD TRANSFUSION DURING THE COVID-19 PANDEMIC

The novel COVID-19 outbreak was reported by the World Health Organization (WHO) on January 30, 2020 and later declared a global pan-demic on 11 March 2020 [20]. The Indian government responded by implementing a state-wide lockdown to stop its spread. Consequently, blood donations were significantly reduced due to travel restrictions and concerns about contracting COVID-19 [21]. However, it was essential to maintain a blood supply in blood banks to meet the needs of patients dependent on transfusion. This includes accident victims, thalassemic patients, severely ill people, and pregnant women. Thus, the outbreak of COVID-19 and its containment measures presented blood centres with a serious challenge in balancing blood demand and supply and devising preparedness [22,23]. Blood transfusions from COVID-19-positive donors have not yet been reported to transmit infection, but active vigilance is essential to prevent transmission [24-26]. A new set of guidelines for safe blood transfusion procedure was released by the NBTC in June 2020 to combat the COVID-19 pandemic. The Haemovigilance Programme of India (HvPI) has enrolled 391 more blood banks during the pandemic, reaching 1190 out of 2760 licensed blood banks in the country to implement these guidelines at the national level. To maintain safety, the NBTC in its guidelines urged blood banks and camp organisers to exclude donors who are in the high-risk.

## CURRENT CHALLENGES

Blood transfusion safety and quality are at risk in many countries due to a lack of effective haemovigilance programs. Despite an increase in reports, challenges and barriers remain to implementing health surveillance systems, especially in developing countries. Blood transfusion safety and quality are at risk in many countries due to a lack of effective haemovigilance programs. Despite an increase in reports, challenges and barriers remain to implementing health surveillance systems, especially in developing countries. In implementing a haemovigilance system, the following challenges must be overcome: i) fragmented and disorganized blood transfusion and health systems, ii) lack of understanding/ awareness of haemovigilance among clinicians and health workers, iii) shortage of experts/expertise on haemovigilance, iv) absence of computerized management system and use of software, v) absence of well-defined haemovigilance structure and protocols, vi) lack of knowledge about adverse event reporting, vii) failure to recognise adverse events, viii) lack of a regulatory framework for haemovigilance, ix) lack of transparency and confidence in government agencies, x) lack of government commitment, xi) fear of retribution and punishment, xii) low participation rate, xiii) health systems, team members, or departmental inconsistencies, xiv) and lack of clarity regarding the relationship between the transfusion and the reaction. Furthermore, it is difficult to not only detect cases, but also get the right information. Communication with blood banks, both governmental and private, is difficult. In the future, hospitals should be motivated to notify patients about transfusion events, maintain functional transfusion committees and improve data analysis and donor monitoring.

## FORTHCOMING EXPANSIONS

A safe and adequate supply of blood is a fundamental need of healthcare. There are two major challenges facing the country today: a lack of blood supply and transfusion-transmitted infections (TTIs). The WHO estimates that India still lacks approximately 1.95 million units of blood per year. A study by the National AIDS Control Organization found 1342 Indians acquired human immunodeficiency virus through transfusions in 2018-2019, raising serious safety and quality concerns. We still have a long way to go to ensure patients have access to safe and adequate blood after COVID-19, which has further exacerbated this issue. The blood transfusion service is disorganised and fragmented at the operational level, making it difficult for the blood banks and end users to communicate effectively, resulting in an incoherent demand and supply gap, which ultimately affects the quality and availability of blood. Since blood-borne diseases are rare, 1% of the healthy population is enough to ensure a safe blood supply in India. However, people are not aware of the importance of donating blood voluntarily, and despite being the second most populous nation in the world, we are unable to reach this goal. India aims to achieve Universal Health Coverage by 2030 through programmes such as Ayushman Bharat, National Health Policy, and the National Digital Health Mission. Guidelines [3,4,27], that may prove helpful in achieving the health mission in the future (**Table 1**). By implementing these suggestions, India can accomplish its goal of achieving universal health cover by fighting against all possible infectious and non-infectious complications.

**Table 1:** Recommendations for improving the haemovigilance system in India

Implementation of guidelines	Utilization of technology for improved diagnosis, treatment and safety	Awareness
Adaptation of haemovigilance at blood transfusion centres across the country.	Equipment for blood handling, administration and storage should be improved on regular basis.	Health bulletin for haemovigilance should be published on regular basis.
All the blood banks should be forced to follow standard operating protocol under D&C Act.	Technology-based improvements in transfusion reaction diagnosis and reporting should be implemented.	Clinicians should be trained to report transfusion reactions as per guidelines.
Blood transfusion guidelines should be updated regularly by the CDSCO based on the TRRF.	In all hospital settings, IgA levels, leukoreduced WBCs, and improved bacteriological testing techniques should be used to minimise transfusion-transmitted infectious risks.	Training for health practitioners and staff in the handling, administration and storage of blood components should be provided on a regular basis.
Blood donation centres should implement safety measures to prevent the spread of COVID-19 to their donors and recipients.	Advanced techniques for preparing blood components (such as PRBCs, PLTC, RDP, and SDP) should be made available to more centres because improved immune haematological techniques can reduce immune-mediated transfusion reactions.	
The blood bank and treating clinician should be notified immediately of any suspected reactions		

D&C – drugs and cosmetics, CDSCO – Central Drug Standard Control Organization, TRFF – transfusion reaction reporting form, IgA – immunoglobulin A, WBC – white blood cell, PRBC – packed red blood cell, PLTC – platelet count, RDP – random donor platelet, SDP – single donor platelets

## CONCLUDING REMARKS

Blood transfusions were negatively affected by the COVID-19 pandemic worldwide. Blood transfusions are a possible way for this virus to spread. For public confidence in blood safety and supply, blood systems need to adopt a national approach to coherence and coordination in order to bolster public confidence in blood safety and supply. The current COVID-19 pandemic exacerbated an already challenging situation, and haemovigilance is the only way to avoid possible transfusion-transmission risks. Although India's haemovigilance reporting trend has increased, there remain wide disparities in reporting rates, reporting quality, and frequency. Implementation of COVID-19 preventive measures helps in the safety of blood donor and blood transfusion services. Further study is required on transfusion responses in COVID-19-positive individuals.
